# Effect of immunosuppression on the human mesangial cell cycle

**DOI:** 10.3892/mmr.2014.2861

**Published:** 2014-11-04

**Authors:** XIAOSHUANG ZHOU, BIRUH WORKENEH, ZHAOYONG HU, RONGSHAN LI

**Affiliations:** 1 Department of Nephrology, Provincial People’s Hospital of Shanxi Medical University, Taiyuan, Shanxi 030001, P.R. China; 2 Department of Nephrology, Baylor College of Medicine, Houston, TX 77030, USA; 3 Department of Nephrology, Shanxi Provincial People’s Hospital, Taiyuan, Shanxi 030001, P.R. China

**Keywords:** cell cycle, mesangial cell, calcineurin-inhibitors, methylprednisolone, mycophenolic acid

## Abstract

The present study investigated the effects of immunosuppressive agents [tacrolimus (Tac), cyclosporine A (CsA), mycophenolic acid (MMF) and methylprednisone (MP)] on the proliferation, cell cycle progression and apoptotic rate of human mesangial cells. Cultured human mesangial cells were treated with several concentrations of the immunosuppressive agents for 24, 48 or 72 h. Cell cycle progression, proliferation and apoptosis were analyzed using an MTT assay and flow cytometry. Tac and CsA significantly inhibited the proliferation of human mesangial cells in a dose- and time-dependent manner. Cell cycle analysis revealed that Tac and CsA arrested mesangial cells in the G_0_/G_1_ phase, preventing them from entering S phase. Similarly, MP inhibited human mesangial cell growth by causing cell cycle arrest in G_0_/G_1_ phase. MMF also inhibited mesangial cell proliferation, but accomplished this by preventing progression from S phase to the G_2_/M phase. The combination of MP and MMF synergistically inhibited mesangial cell proliferation. Tac, CsA, MP and MMF inhibited proliferation of human mesangial cells by blocking progression of the cell cycle. In conclusion, these agents, sequentially or in combination, may be used to effectively treat mesangial proliferative glomerular disease.

## Introduction

Several glomerular diseases, including focal segmental glomerulosclerosis (FSGS) variants, immunoglobulin A (IgA)nephropathy and lupus nephritis are associated with mesangial cell proliferation and expansion (1). Thus, immunosuppressive agents that have an inhibitory effect on mesangial cell expansion and proliferation are of considerable interest. Patients with glomerulonephritis involving mesangial proliferation are often treated with agents including corticosteroids, calcineurin-inhibitors, cyclophosphamide (CyA) and anti-metabolites such as mycophenolic acid (MMF). These agents have narrow therapeutic windows and serious side-effects (2,3). Combination and sequential therapy using various immunosuppressive agents have been used to successfully treat kidney transplant recipients and myelogenous leukemia patients (4–6). Therefore, it was hypothesized that a complementary or sequential immunosuppressant treatment strategy may be capable of effectively suppressing human mesangial cell proliferation. The aim of the present study was to acquire more information regarding the effects of these immunosuppressive agents on the cell cycle progression of human mesangial cells and to investigate whether a combination of these agents may result in a more effective suppression of mesangial cell proliferation.

Inflammation or cell injury triggers mesangial cell proliferation, which causes activation and progression of the cell cycle. Interfering with processes at any stage of the cell cycle can arrest proliferation or promote apoptosis (7). Drugs commonly used to treat glomerulonephritis include tacrolimus (Tac), cyclosporine A (CsA), methylprednisone (MP) and MMF. Several studies have demonstrated that these agents can inhibit the proliferation of mesangial cells and may therefore be effectively used to treat glomerular disease (1,8–11). However, a detailed explanation regarding the effect that these drugs exert on the human mesangial cell cycle is lacking. Knowledge of the mechanism of the effects of these drugs on the cell cycle is of potential use in disease monitoring and treatment of glomerular disorders. The present study investigated how each of these agents influenced the proliferation, apoptosis and cell cycle progression of human mesangial cells using a dose-escalation and sequential approach.

## Materials and methods

### Cell cultures

A human mesangial cell line T-SV40, provided by Dr Li Xuewang at Peking Union Medical College Hospital (Beijing, China) (12,13), was cultured at 37°C in a humidified 5% CO_2_ atmosphere with RPMI-1640 medium (Sigma, St. Louis, MO, USA) containing 10% fetal calf serum (FCS; Sijiqing Biological Engineering Materials Co., Ltd., Hangzhou, China). Prior to stimulating proliferation, 60%-confluent cells were starved in serum-free medium for 24 h and then treated with medium containing 10% FCS and various immunosuppressive agents. Cells were used at passage 17 and no mycoplasmic infection was detected.

### MTT assay

Human mesangial cells were seeded at a density of 1×10^5^/ml into 96-well plates for 24 h. Each plate contained three wells of each experimental condition and three control wells. Following treatment with various immunosuppressive agents for 24, 48 or 72 h, cells were incubated with MTT (0.5%, Sigma) for 4 h at 37°C. The medium was subsequently removed and 150 μl dimethyl sulfoxide (Sigma-Aldrich, Beijing, China) was added to each well prior to measuring the absorbance (490 nm, model 550, Bio-Rad, Hercules, CA, USA).

### Cell cycle analysis

Cell cycle progression was assessed by flow cytometry (FCM). Human mesangial cells were seeded at a density of ~1×10^5^/ml in six-well plates for 24 h prior to the addition of various immunosuppressive agents, including TAC, CsA, MP and MMF (all Sigma-Aldrich) for 24, 48 or 72 h. Cells were collected, fixed in 1% methanol-free formaldehyde (Sigma-Aldrich) for 20 min and suspended in 70% ethanol solution to dehydrate for 24 h at −20°C. Cells were washed with phosphate-buffered saline (PBS; Sigma-Aldrich) and incubated in PBS containing RNAse for 10 min at room temperature. Finally, 200 μl propidium iodide solution was added to each well for 10 min on ice to stain the nuclei. Samples were immediately examined by FCM using a FACstar Plus cytometer (Becton-Dickinson, Mountain View, CA, USA) and the results analyzed by Cell Quest software (Becton-Dickinson). Each experiment was performed three times, and the ratio of cells in the G_0_/G_1_, S and G_2_/M phases was determined and expressed as the mean ± standard deviation (SD).

### Cell apoptosis analysis

Apoptotic cells were detected by FCM. Human mesangial cells were seeded at a density of ~1×10^5^/ml in six-well plates for 24 h. Following administration of various immunosuppressive agents for 24, 48 or 72 h, cells were collected, washed with PBS and adjusted to a density of ~1×10^6^/ml with PBS. 100 μl cell suspension was transferred into tubes containing 5 μl Annexin V/fluorescein isothiocyanate (Life Technologies, Grand Island, NY, USA) and 10 μl propidium iodide solution. The cells were fixed for 15 min in the dark. Finally, 400 μl PBS was added to each tube and the contents immediately analyzed with the flow cytometer (Becton-Dickinson) to detect apoptosis.

### Statistical analysis

All experiments were repeated three times and results were presented as the mean ± SD. The treatment effects were analyzed by one-way analysis of variance using Sigma stat 3.5 (Systat Software, San Jose, CA, USA) to test differences amongst the groups. P<0.05 was considered to indicate a statistically significant difference between values.

## Results

### Tacrolimus

**Figure 1:**
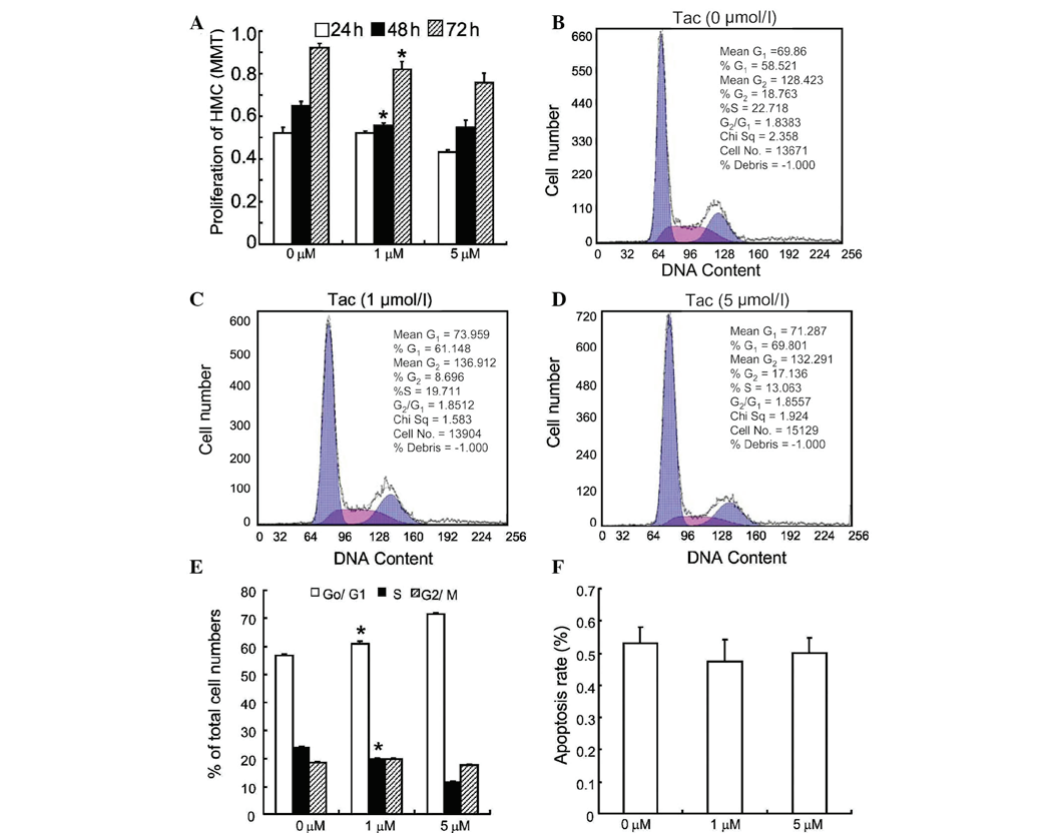
Tac prevents progression of the cell cycle of HMCs from G_0_/G_1_ to S phase. (A) Quiescent HMCs were treated with 10% fetal calf serum in the absence or presence of Tac (1 and 5 μmol/l) and their proliferation was assessed via MTT assay at 24, 48 and 72 h (^*^P<0.05 vs. 0 μmol/l). (B–D) Cell cycle progression of HMCs in response to various concentrations of Tac was analyzed by flow cytometry 48 h following treatment. (E) Statistical analysis indicated that upon exposure to 1 and 5 μmol/l Tac for 48 h, the percentage of HMCs in the S phase and G_0_/G_1_ phase was significantly altered (^*^P<0.01 vs. 0 μmol/l). (F) HMCs were treated with Tac (1 and 5 μmol/l) for 48 h and the apoptotic rate was assessed by flow cytometry. All values are presented as the mean ± standard deviation values of three independent experiments. Tac, tacrolimus; HMC, human mesangial cell.

The effects of Tac on the cell cycle of human mesangial cells were examined, firstly by treating human mesangial cells with Tac (1–5 μmol/l) and assessing their proliferation by an MTT assay. Cellular proliferation was significantly decreased following Tac treatment. This inhibitory effect occurred in a dose- and time-dependent manner (Fig. 1A). The effects of Tac on cell cycle progression were then examined (Fig. 1B–D). Upon exposure to 5 μmol/l Tac for 48 h, the percentage of cells in the S phase decreased by 41%, while the percentage of cells in G_0_/G_1_ phase increased by 30%. These results indicated that Tac prevented the progression of human mesangial cells into S phase (Fig. 1E). The effects of Tac on apoptosis of human mesangial cells were also examined. Tac (at 1 and 5 μmol/l) did not significantly alter the apoptotic rate of human mesangial cells following 48 h of treatment (Fig. 1F).

### Cyclosporine A

**Figure 2:**
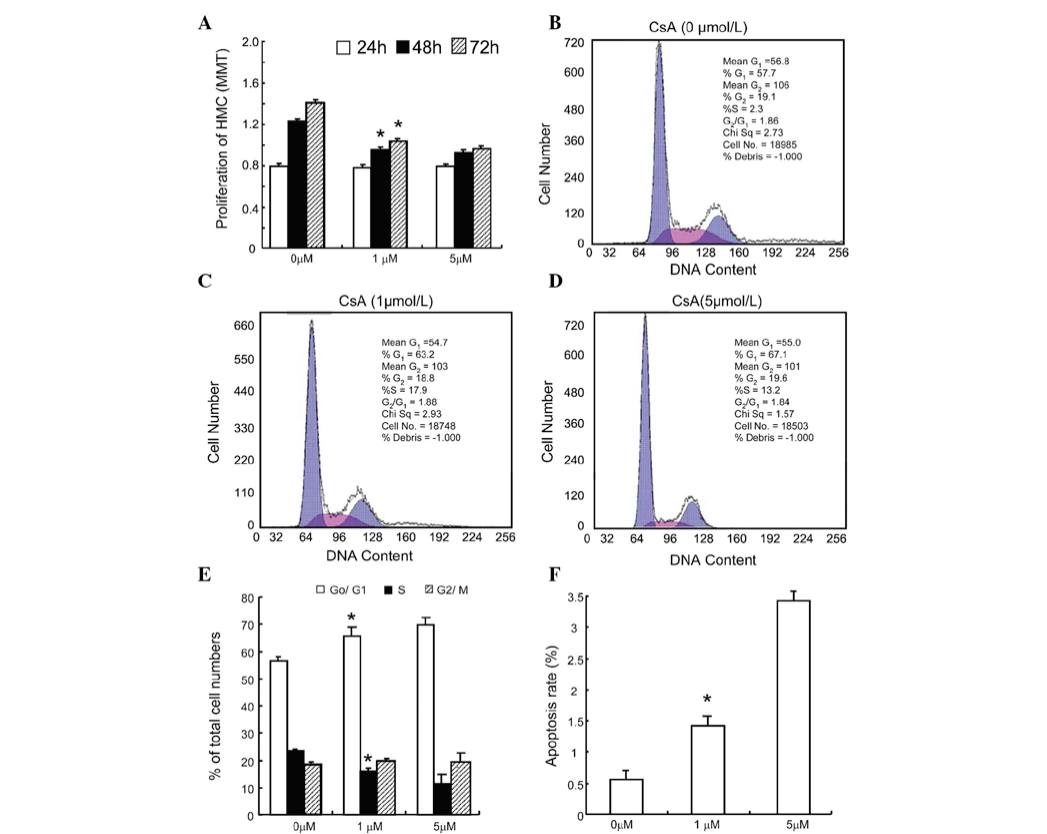
CsA causes HMC growth arrest at the G_0_/G_1_ phase. (A) Quiescent HMCs were stimulated by 10% fetal calf serum with or without CsA (1 and 5 μmol/l) for 24, 48 and 72 h, and proliferation was examined by an MTT assay (^*^P<0.001 vs. 0 μmol/l ). (B–D) Representative flow cytometry results of HMC cycle analysis (control and CsA at 1 and 5 μmol/l for 48 h). (E) Statistical analysis results of B–D (^*^P<0.01 vs. 0 μmol/l). (F) HMCs were treated with CsA (1 and 5 μmol/l) for 48 h and apoptotic rates were measured by flow cytometry (^*^P<0.01 vs. 0 μmol/l). All values are presented as the mean ± standard deviation of three independent experiments. CsA, cyclosporine A; HMC, human mesangial cell.

As in the case of Tac, when human mesangial cells were exposed to CsA (1 and 5 μmol/l) in a dose- and time-dependent manner, cellular proliferation was inhibited (Fig. 2A). Following 48 h of exposure to CsA (1 and 5 μmol/l), the percentage of cells in S phase was significantly decreased and there was a significant increase in the percentage of cells in the G_0_/G_1_ phase (Fig. 2B–D). This indicated that CsA arrested human mesangial cells prior to their entry into S phase (Fig. 2E). Finally, the effects of CsA on apoptosis of human mesangial cells were assessed. When cells were exposed to CsA (1 and 5 μmol/l) for 48 h, the percentage of apoptotic cells significantly increased in a dose-dependent manner (Fig. 2F).

### Methylprednisolone

**Figure 3:**
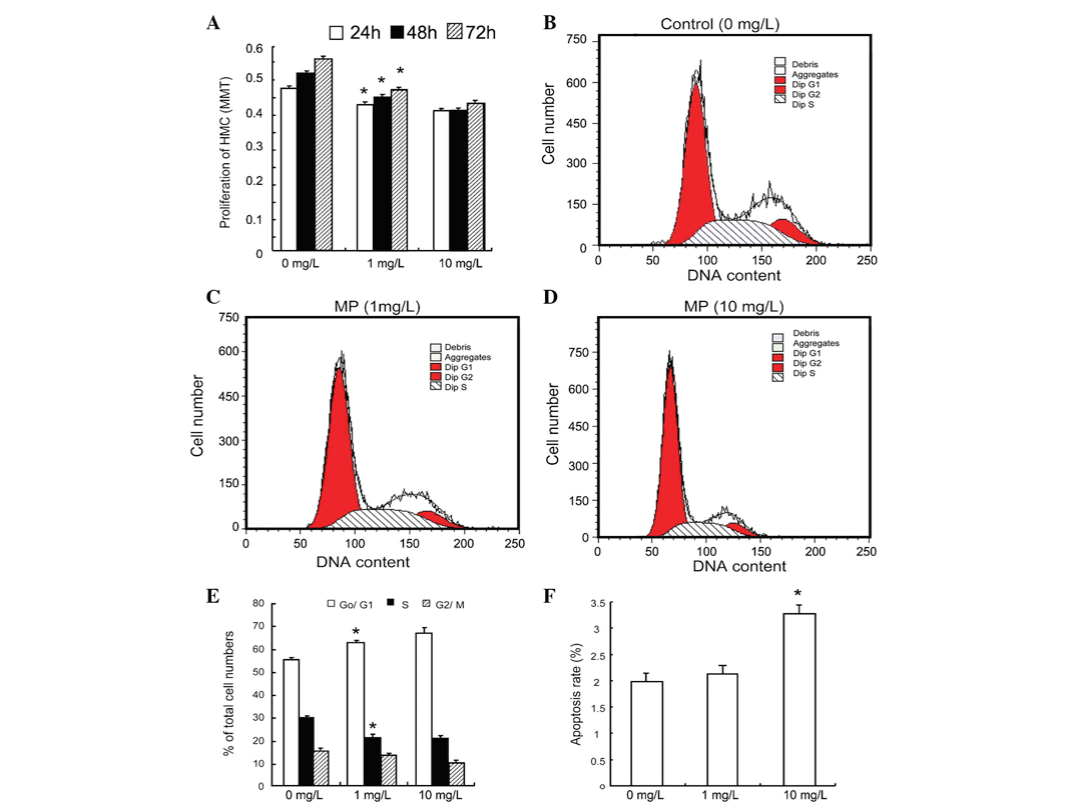
MP prevents HMCs from entering S phase. (A) Quiescent HMCs were treated with 10% fetal calf serum in the absence and presence of MP (1 and 10 mg/l) and subjected to an MTT assay (^*^P<0.05 vs. 0 mg/l). (B–D) At 48 h, the cell cycle progression was assessed by flow cytometry. (E) Statistical analysis of B–D (^*^P<0.05 vs. 0 μmol/l). (F) Statistical analysis of the apoptotic rate assessed by flow cytometry. At 10 mg/l for 48 h, MP significantly increased apoptosis in HMCs (^*^P<0.01 vs. 0 μmol/l). Values are expressed as the mean ± standard deviation of six independent experiments. MP, methylprednisolone; HMC, human mesangial cell.

The influence of MP on human mesangial cell growth has not previously been studied, to the best of our knowledge. At concentrations of 1 and 10 mg/l, MP inhibited the proliferation of human mesangial cells in a dose- and time-dependent manner (Fig. 3A). It was also determined that at concentrations of 1 and 10 mg/l, MP significantly decreased the percentage of cells in S phase, while increasing the percentage of cells in G_0_/G_1_ phase (Fig. 3B–E). Similarly to CsA, MP (1–10 mg/l) significantly increased the apoptotic rate in human mesangial cells following 48 h of treatment (Fig. 3F).

### Mycophenolic acid

**Figure 4:**
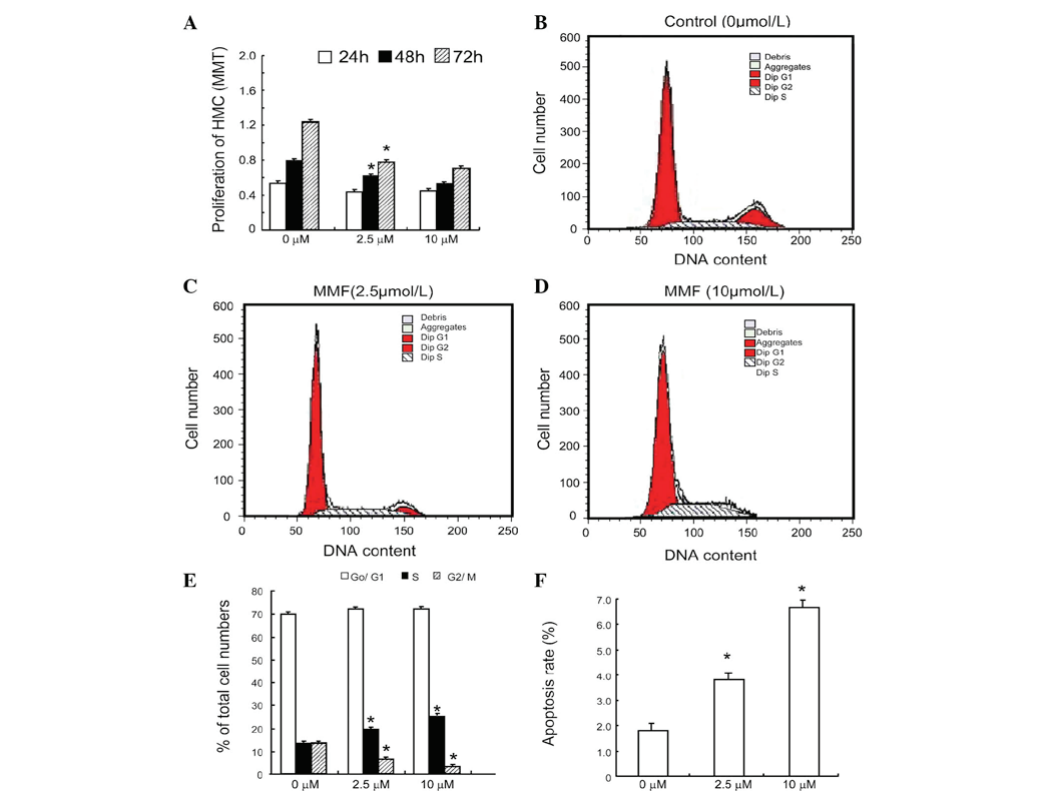
MMF blocks entry of HMCs into G_2_/M phase. (A) Quiescent HMCs were treated with MMF for 24, 48 or 72 h. The proliferative rate was assessed using an MTT assay (^*^P<0.01 vs. 0 μmol/l). (B–D) The cell cycle of HMCs was analyzed by flow cytometry following 48 h of treatment. (E) Statistical analysis of B–D (^*^P<0.01 vs. 0 μmol/l). (F) The apoptotic rate was measured by flow cytometry following 48 h of treatment. MMF significantly increased apoptosis in HMCs (^*^P<0.01 vs. 0 μmol/l). Values are expressed as the mean ± standard deviation of six independent experiments. MMF, mycophenolic acid; HMC, human mesangial cell.

The present study investigated how MMF influenced the proliferation of human mesangial cells and found that 0.25–10 μmol/l MMF significantly inhibited the proliferation of human mesangial cells following 24, 48 or 72 h of treatment (Fig. 4A). MMF also significantly suppressed the entry of cells into G_2_/M phase, causing cell cycle arrest in the S phase (Fig. 4B–E). As shown in Fig. 4F, there was a significant increase in the early apoptotic rate of human mesangial cells that were treated for 48 h with MMF.

### Immunosuppressants inhibit proliferation and cell cycle progression of human mesangial cells

**Figure 5:**
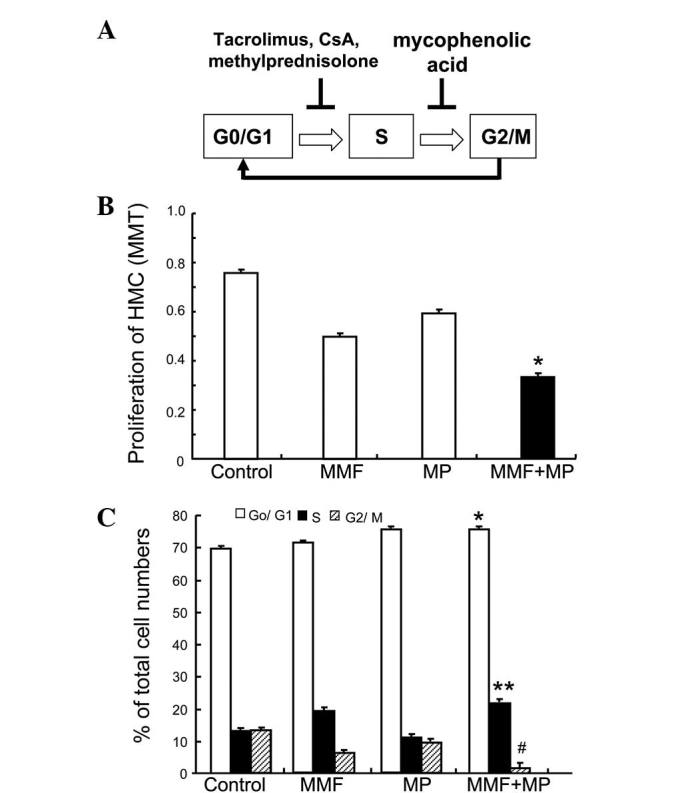
Different immunosuppressive agents target different phases of the cell cycle in HMCs. (A) Schematic displaying the actions of immunosuppressive agents on the cell cycle of HMCs. Calcineurin-inhibitors (Tac and CsA) and MP inhibit HMC proliferation by blocking cell entry into S phase, while MMF blocks cell cycle progression by limiting HMC entry into G_2_/M phase. (B and C) MMF (2.5 mM) and MP (1 mg/l) synergistically inhibited the proliferation of HMCs by interfering with the cell cycle at G_0_/G_1_ phase and S phase (^*^P<0.05 vs. MMF; ^**^P<0.01 vs. MP; ^#^P<0.01 vs. MMF or MP; values are presented as the mean ± standard deviation (n=3). HMC, human mesangial cell; Tac, tacrolimus; CsA, cyclosporine A; MMF, mycophenolic acid; MP, methylprednisone

The effects of Tac, CsA, MP and MMF on human mesangial cell cycle progression are summarized in Fig. 5A. Since MMF is often used with adjunctive immunosuppressants, human mesangial cells were treated with 2.5 mmol/l MMF in order to block cells in the S phase from entering into G_2_/M phase and 1 mg/l MP to block cells progressing from G_0_ phase to S phase. This combination of drugs inhibited the proliferation of mesangial cells more efficiently than each drug separately (Fig. 5B). This combination also interfered with the progression of mesangial cells in the G_0_/G_1_ and S phase (Fig. 5C).

## Discussion

Mesangial cells serve a number of functions in the renal glomerulus, including structural support of the capillary tuft, modulation of glomerular hemodynamics and phagocytic removal of macromolecules and immune complexes. These cells also have complex interactions with infiltrating inflammatory cells, responding and contributing to the amplification of inflammation, fibrosis and the development of glomerulosclerosis (14). The proliferation of mesangial cells is a common pathological feature of glomerular diseases, including IgA nephropathy and lupus nephritis (11). For these reasons, numerous studies have investigated the contribution of mesangial cells to the development of glomerulosclerosis (15). However, these studies have concentrated on cultured cells or animal models of glomerular injury and there have been few studies of human mesangial cells. Specific targeting of mesangial cell proliferation may more effectively retard the progress of glomerular disease.

In mouse renal tubular epithelial cells, CsA caused cell cycle arrest in the G_0_/G_1_ phase and inhibited DNA synthesis (16). These results are similar to those the present study obtained on human mesangial cells. Compared to Tac, CsA caused a marked increase in apoptosis in human mesangial cells. This response may be linked to the activation of pre-apoptotic pathways or to the release of cytochrome c into the cytosol (17–20).

The present study found that similarly to Tac and CsA, MP caused mesangial cell cycle arrest in the G_0_/G_1_ phase and prevented cells from entering the S phase. This is in agreement with a study by Bladh *et al* (21), who reported that glucocorticoids can decrease the percentage of cells in S/G_2_/M phase and impair the proliferation of human embryonic kidney 293 cells by suppressing nuclear factor κ-light-chain-enhancer of activated B-cell activity. Glucocorticoids exert an antiproliferative effect in numerous cell types (22–26); therefore, it was hypothesized that the anti-proliferative effect of MP may be due to induction of cyclin-dependent kinase inhibitors such as p21Cip1 or p57Kip2 (27,28). Alternatively, MP may suppress c-myc or cyclins, which are capable of stimulating cell cycle progression (19). In contrast to Tac, CsA and MP, MMF significantly inhibited mesangial cell growth by preventing cells from entering G_2_/M phase. This increased the percentage of cells in the S phase and decreased the percentage of cells in G_2_/M phase.

The present study suggested a theoretical basis for sequential therapy with various immunosuppressive agents to treat glomerular diseases featuring mesangial proliferation. Sequential therapy with various immunosuppressive agents may limit the complications associated with steroid treatment or dependency and potentially provide an alternative treatment for steroid-resistant disease. It was found that the combination of MP and MMF was more effective at inhibiting mesangial cell proliferation.

In conclusion, Tac, CsA, MP and MMF suppressed human mesangial cell proliferation by targeting different phases of the cell cycle. A sequential therapy based on these differences may potentially be used as a strategy to treat proliferative glomerular diseases. Further studies to assess the *in vivo* responses of human mesangial cells to sequential therapy in mesangioproliferative disease models are required.

## References

[1] Akool el-S, Doller A, Babelova A, Tsalastra W, Moreth K, Schaefer L, Pfeilschifter J, Eberhardt W (2008). Molecular mechanisms of TGF beta receptor-triggered signaling cascades rapidly induced by the calcineurin inhibitors cyclosporin A and FK506. J Immunol.

[2] Trachtman H, Vento s, Gipson D (2011). Novel therapies for resistant focal segmental glomerulosclerosis (FONT) phase II clinical trial: study design. BMC Nephrol.

[3] Ponticelli C, Passerini P (2003). Other immunosuppressive agents for focal segmental glomerulosclerosis. Semin Nephrol.

[4] Sabuda-Widemann D, Grabensee B, Schwandt C, Blume C (2009). Mycophenolic acid inhibits the autocrine PDGF-B synthesis and PDGF-BB-induced mRNA expression of Egr-1 in rat mesangial cells. Nephrol Dial Transplant.

[5] Radeke HH, Kuster S, Kaever V, Resch K (1993). Effects of cyclosporin and FK-506 on glomerular mesangial cells. Evidence for direct inhibition of thromboxane synthase by low cyclosporin concentrations. Eur J Clin Pharmacol.

[6] Miao L, Sun J, Yuan H, Jia Y, Xu Z (2006). Combined therapy of low-dose tacrolimus and prednisone in nephrotic syndrome with slight mesangial proliferation. Nephrology (Carlton).

[7] Pastukhov O, Schwalm S, Römer I, Zangemeister-Wittke U, Pfeilschifter J, Huwiler A (2014). Ceramide kinase contributes to proliferation but not to prostaglandin E2 formation in renal mesangial cells and fibroblasts. Cell Physiol Biochem.

[8] Anil KMS, Irfan SM, Ranganna K, Malat G, Sustento-Reodica N, Kumar AM, Meyers WC (2008). Comparison of four different immunosuppression protocols without long-term steroid therapy in kidney recipients monitored by surveillance biopsy: five-year outcomes. Transpl Immunol.

[9] Boletis J, Balitsari A, Filiopoulos V, Stamataki E, Lionaki S, Zavos G, Kostakis A (2005). Delayed renal graft function: the influence of immunosuppression. Transplant Proc.

[10] Ren H, Guo N, Lu D (2001). Successful engraftment of HLA-identical sibling cord blood transplantation in an adult with chronic myelogenous leukemia. Chinese Journal of Hematology.

[11] Kurogi Y (2003). Mesangial cell proliferation inhibitors for the treatment of proliferative glomerular disease. Med Res Rev.

[12] Delarue F, Virone A, Hagege J, Lacave R, Peraldi MN, Adida C, Rondeau E, Feunteun J, Sraer JD (1991). Stable cell line of T-SV40 immortalized human glomerular visceral epithelial cells. Kidney Int.

[13] Ruan XZ, Varghese Z, Fernando R, Moorhead JF (1998). Cytokine regulation of low-density lipoprotein receptor gene transcription in human mesangial cells. Nephrol Dial Transplant.

[14] Pereira RL, Felizardo RJ, Cenedeze MA (2014). Balance between the two kinin receptors in the progression of experimental focal and segmental glomerulosclerosis in mice. Dis Mod Mech.

[15] Liu CY, Zhou LL, Cheng Q, Jiang SN, Sheng J, Sun JD, Zhao JY (2014). Effect of bradykinin on renal mesangial cell proliferation and extracellular matrix secretion. Genet Mol Res.

[16] Jennings P, Koppelstaetter C, Aydin S, Abberger T, Wolf AM, Mayer G, Pfaller W (2007). Cyclosporine A induces senescence in renal tubular epithelial cells. Am J Physiol Renal Physiol.

[17] Choi SJ, You HS, Chung SY (2008). Tacrolimus-induced apoptotic signal transduction pathway. Transplant Proc.

[18] Migita K, Eguchi K (2001). FK 506-mediated T-cell apoptosis induction. Transplant Proc.

[19] Park JW, Bae EH, Kim IJ, Ma SK, Choi C, Lee J, Kim SW (2010). Paricalcitol attenuates cyclosporine-induced kidney injury in rats. Kidney Int.

[20] de Arriba G, de Hornedo JP, Rubio SR, Fernández MC, Martinez SB, Camarero MM, Cid TP (2009). Vitamin E protects against the mitochondrial damage caused by cyclosporin A in LLC-PK1 cells. Toxicol Appl Pharmacol.

[21] Bladh LG, Lidén J, Pazirandeh A, Rafter I, Dahlman-Wright K, Nilsson S, Okret S (2005). Identification of target genes involved in the antiproliferative effect of glucocorticoids reveals a role for nuclear factor-(kappa)B repression. Mol Endocrinol.

[22] Rogatsky I, Trowbridge JM, Garabedian MJ (1997). Glucocorticoid receptor-mediated cell cycle arrest is achieved through distinct cell-specific transcriptional regulatory mechanisms. Mol Cell Biol.

[23] Smith E, Redman RA, Logg CR, Coetzee GA, Kasahara N, Frenkel B (2000). Glucocorticoids inhibit developmental stage-specific osteoblast cell cycle. Dissociation of cyclin A-cyclin-dependent kinase 2 from E2F4-p130 complexes. J Biol Chem.

[24] Rhee K, Reisman D, Bresnahan W, Thompson EA (1995). Glucocorticoid regulation of G1 cyclin-dependent kinase genes in lymphoid cells. Cell Growth Differ.

[25] Helmberg A, Auphan N, Caelles C, Karin M (1995). Glucocorticoid-induced apoptosis of human leukemic cells is caused by the repressive function of the glucocorticoid receptor. EMBO J.

[26] Sánchez I, Goya L, Vallerga AK, Firestone GL (1993). Glucocorticoids reversibly arrest rat hepatoma cell growth by inducing an early G1 block in cell cycle progression. Cell Growth Differ.

[27] Corroyer S, Nabeyrat E, Clement A (1997). Involvement of the cell cycle inhibitor CIP1/WAF1 in lung alveolar epithelial cell growth arrest induced by glucocorticoids. Endocrinology.

[28] Cha HH, Cram EJ, Wang EC, Huang AJ, Kasler HG, Firestone GL (1998). Glucocorticoids stimulate p21 gene expression by targeting multiple transcriptional elements within a steroid responsive region of the p21waf1/cip1 promoter in rat hepatoma cells. J Biol Chem.

